# Association of Maternal Prepregnancy Weight and Gestational Weight Gain With Children’s Allergic Diseases

**DOI:** 10.1001/jamanetworkopen.2020.15643

**Published:** 2020-09-02

**Authors:** Yiting Chen, Jianzhen Zhu, Jiajun Lyu, Yuanqing Xia, Yong Ying, Yabin Hu, Jiajie Qu, Shilu Tong, Shenghui Li

**Affiliations:** 1 School of Medicine, Shanghai Jiao Tong University, Shanghai, China; 2 School of Public Health, Shanghai Jiao Tong University, Shanghai, China; 3 School of International and Public Affairs, Shanghai Jiao Tong University Shanghai, China; 4 School of Medicine, Shanghai Jiao Tong University Shanghai, China; 5 Shanghai Municipal Education Commission, Shanghai, China; 6 Institute of Environment and Population Health, Anhui Medical University School of Public Health, Hefei, China; 7 Queensland University of Technology School of Public Health and Social Work, Brisbane, Queensland, Australia; 8 MOE-Shanghai Key Laboratory of Children’s Environmental Health, Department of Public Health, School of Medicine, Shanghai Jiao Tong University, Shanghai, China

## Abstract

**Importance:**

Information on the association between maternal prepregnancy body weight, gestational weight change, and childhood allergies is not consistent. Little is known on whether there is a combined association with comprehensive childhood allergies.

**Objective:**

To examine the association of maternal prepregnancy body mass index and gestational weight gain (GWG) with the risk of childhood allergic diseases including asthma and/or wheezing, allergic rhinitis, eczema, and food and/or drug allergy.

**Design, Setting, and Participants:**

This cross-sectional study was conducted from April 12 to June 1, 2019, using the Shanghai Children Allergy Study (SCAS), a large, population-based survey. A multistage and multistrata sampling approach was applied to conduct the survey in 13 districts across Shanghai, China, with an enrollment of 15 145 mother-child pairs.

**Exposures:**

Reported maternal weight before pregnancy and at the time of delivery were requested in the survey; GWG was examined in absolute terms and with reference to the Institute of Medicine guideline.

**Main Outcomes and Measures:**

The ascertainment of allergic diseases was based on the International Study of Asthma and Allergies in Childhood questionnaire.

**Results:**

Among 15 145 children aged 3 to 14 years (7911 [52.2%] boys) within the SCAS, 8877 children (58.6%) were screened for allergic diseases. Multivariable log-binomial regression models suggested that excessive GWG was associated with risks of 19% for asthma/wheezing, 11% for allergic rhinitis, and 10% for eczema in the children. Gestational weight gain extremely above the Institute of Medicine guideline in women who were overweight/obese before pregnancy was associated with the highest risk of childhood asthma/wheezing (adjusted prevalence ratio, 1.42; 95% CI, 1.16-1.74; *P* = .001), allergic rhinitis (adjusted prevalence ratio, 1.32; 95% CI, 1.12-1.56; *P* = .001), and eczema (adjusted prevalence ratio, 1.24; 95% CI, 1.08-1.41; *P* = .002). Gestational weight gain below the Institute of Medicine guideline was associated with an attenuated risk of 13% for childhood asthma/wheezing, 11% for allergic rhinitis, 14% for eczema, and 15% food/drug allergy when mothers were of prepregnancy normal weight; similar associations were observed in underweight mothers, but there was no association in overweight mothers. Generally, the associations were similar in the stratified analysis according to children’s age group. Moreover, the results were similar in the allergen test subgroup.

**Conclusions and Relevance:**

The findings of this study suggest that excessive GWG may be a risk factor for childhood allergic diseases. Further studies on the long-term effects of the intrauterine environment on children’s health may take maternal weight management during pregnancy into consideration since GWG could be a controllable and modifiable risk factor.

## Key Points

**Question:**

Are maternal prepregnancy weight and gestational weight gain associated with childhood allergies?

**Findings:**

In this cross-sectional study evaluating 8877 children, excessive gestational weight gain was associated with a risk of asthma and/or wheezing, allergic rhinitis, eczema, and food and/or drug allergy, and this risk appeared to be higher when coexisting with maternal prepregnancy overweight or obesity. Low gestational weight gain was associated with a reduced risk of allergies in children of women with low or normal prepregnancy weight, while there was no association in women who were overweight or obese.

**Meaning:**

The findings of this study suggest that, to meet the challenge of increasing childhood allergies, the management of prepregnancy weight and gestational weight gain must be considered.

## Teaser

This cross-sectional study examines the incidence of childhood allergies in women with varying levels of prepregnancy weight and gestational weight gain.

## Introduction

Shanghai, China, is a fast-growing city with rapid urbanization that had brought about great changes on lifestyle, accompanied by an increasing prevalence of obesity in the general population as well as in pregnant women.^1^ In 2009, the Institute of Medicine (IOM) published a guideline recommending the appropriate gestational weight gain range for pregnant women in the US.^2^ A national survey conducted from 2010 to 2011 revealed that, among US women, only 32% met the IOM standard; 47% exceeded the proper range, defined as excessive gestational weight gain (GWG).^3^ A rapid increase in the prevalence of childhood allergic diseases has also attracted global attention.^4^ The prevalence of childhood allergic diseases increased nearly 5-fold in the period from 1990 to 2011 in China.^5^ A 10-city survey illustrated that Shanghai had the highest prevalence of childhood allergic diseases except wheezing.^6^ Data have suggested that, although allergic diseases can begin in early childhood, they generally persist into adulthood and result in morbidity throughout life.^7^

In the developmental origins of health and disease theory,^8,9,10,11,12,13^ childhood allergic diseases are considered to originate in the periconceptional period.^14^ Maternal prepregnancy body mass index (BMI) and excessive GWG during pregnancy can alter the intrauterine environment, which may affect the development of the immune system of the fetus, thus leading to increased susceptibility to allergic diseases.^15^ Increased risk of childhood allergic diseases associated with high maternal BMI before pregnancy has been suggested in several cohort studies.^8,9,10,16,17,18,19,20,21,22,23,24,25^ In contrast, associations of GWG with childhood allergic diseases have received less attention.^8,10,17,21,24,25,26^ A meta-analysis analyzed the association between maternal BMI and childhood allergic diseases using data from 14 studies (including 108 321 mother-child pairs); however, only 3 of these studies simultaneously examined maternal GWG, with findings suggesting that excessive GWG could be an independent risk factor for childhood wheezing or asthma.^8^ By contrast, an association between low GWG and childhood allergies was not found. However, 2 cohort studies from the US suggested that low GWG was associated with an increased risk of childhood asthma.^17,25^ In addition, to our knowledge, only one study focused on dermatitis, suggesting that GWG was associated with childhood susceptibility to allergic dermatitis.^24^ Owing to limited evidence, the possible role of maternal weight and weight gain during pregnancy in development of childhood allergic diseases is unclear. Furthermore, almost all of the studies were conducted in high-income countries, which raised the question of whether there are differences in China, the largest low-income country in the world.

We performed a large, cross-sectional study including 31 kindergartens and 17 primary schools covering 13 districts across Shanghai to examine whether maternal prepregnancy BMI and GWG were associated with childhood allergic diseases, including asthma and/or wheezing, allergic rhinitis, eczema, and food and/or drug allergies. We also analyzed the results in children who have taken part in allergen tests (allergen test subgroup). We hypothesized that both excess GWG and low GWG are associated with the development of childhood allergic diseases, and maternal prepregnancy overweight or obesity would be associated with excessive GWG.

## Methods

### Study Design and Population

The Shanghai Children Allergy Study was conducted to investigate the prevalent features of and factors associated with childhood allergic diseases in Shanghai. Ethical approval was granted by the ethics committee of Shanghai Jiao Tong University School of Medicine. Written informed consent was obtained after the purpose of this study was conveyed to parents. Participants did not receive financial compensation. This study followed the Strengthening the Reporting of Observational Studies in Epidemiology (STROBE) reporting guideline for cross-sectional studies.

From April 12 to June 1, 2019, the study was performed using a multistage and multistrata sampling approach. Overall, 7 urban areas (Xuhui, Huangpu, Hongkou, Putuo, Changning, Yangpu, and Pudong New area) and 6 suburban/rural areas (Minhang, Jinshan, Qingpu, Songjiang, Baoshan, and Chongming) were randomly sampled among a total of 9 urban areas and 8 suburban/rural areas in Shanghai. In these areas, 31 kindergartens and 17 primary schools were randomly sampled. The purpose of the investigation was explained to the school and kindergarten principals and teachers in advance.

### Variables

The International Study of Asthma and Allergies in Childhood (ISAAC) questionnaire, which is commonly used among preschool and school-aged children, was adopted to evaluate childhood allergic diseases. Briefly, information both on ever-diagnosed diseases and current symptoms was obtained to identify childhood asthma/wheezing, allergic rhinitis, and eczema, and the determination of food/drug allergy was based mainly on questions related to physician diagnosis. More detailed information on each allergic disease is described in the eAppendix in the Supplement.^27,28,29,30^ The Cronbach α coefficient of the ISAAC allergic questionnaire in the sampled children was 0.94. Validity determined by the Kaiser-Meyer-Olkin method was 0.94.

Information on children’s allergen tests was acquired through parental answer to the question, “Has your child ever had allergen tests?” If the answer was positive, that question was followed by, “What kinds of allergen tests were performed?” The options included immunoglobulin E, skin testing, and others. A further question was, “Were the allergen tests positive or not positive?” If parents stated the test results were positive, they were asked to report the specific allergen identified, including inhaled allergens (eg, animal dander, mugwort, dust mites, mold, and ragweed) and food allergens (eg, dairy products, eggs, seafood, peanuts, wheat, and soy products). Overall, 3229 of 15 145 children who been tested for allergens had positive test results, and 1520 children had negative test results.

Information on maternal height and weight before pregnancy was obtained from the questionnaire and maternal prepregnancy BMI was calculated as weight in kilograms divided by height in meters squared.^31^ Based on World Health Organization guidelines modified for Asian people, mothers who participated in the survey were categorized into 4 groups: underweight (BMI, <18.5), normal weight (BMI, 18.5-22.9), overweight (BMI, 23.0-24.9), and obese (BMI, ≥25.0).^32,33^

Maternal weight at delivery was also documented, and GWG was calculated as maternal weight before delivery minus maternal prepregnancy weight. The analysis of GWG was performed in 2 steps. First, we divided data on GWG into 4 categories (<10.0, 10.0-15.0, 15.0-25.0, and ≥25.0 kg).^17,21^ Second, recommendation of the IOM was applied.^2^ Based on maternal prepregnancy BMI, participants were categorized as weight gained below, above, or within the IOM guideline (12.5-18.0 kg for underweight, 11.5-16.0 kg for normal weight, 7.0-11.5 kg for overweight, and 5.0-9.0 kg for obese). Those whose GWG was above the IOM guideline were further divided into 2 groups; the median from the threshold to the highest value was regarded as the cutoff. One group, from threshold to median, was defined as mildly above the IOM guideline. The other group, from median to the maximum, was defined as extremely above the IOM guideline.

Maternal and paternal characteristics included maternal age at delivery (≤24, 25-29, and ≥30 years), paternal age at delivery (≤24, 25-34, and ≥35 years), family income (<¥6000 and ≥¥6000 [¥1 is equivalent to $1]), parity (≤1 and ≥2), maternal smoking during pregnancy (yes/no), maternal alcohol intake during pregnancy (yes/no), paternal smoking during pregnancy (yes/no), and family history of allergic disease (yes/no).

### Statistical Analysis

Statistical analysis was conducted using the percentage for categorical variables. The χ^2^ tests were applied to compare differences between groups. Univariate and multivariable log-binomial regression models were implemented to calculate the prevalence ratios (PRs) and 95% CIs, assessing the association of maternal prepregnancy BMI and maternal GWG with childhood allergic diseases.^34^ *P* values for trend were assessed by entering the prepregnancy BMI and GWG as continuous variables. Code 1 was assigned for children with positive screening for any of the 4 allergies and 0 for children without any screened allergy. For individual allergic diseases, 1 was coded for children with a target single allergic disease and 0 was used for those without any screened allergy.

Stratified analyses were performed by BMI and the children’s age group, and test for interaction was conducted in multivariable models to investigate possible effect modification by prepregnancy BMI in the association between maternal GWG and childhood allergies.

Sensitivity analyses were performed in children within the allergen test subgroup. Children with positive responses to both the questionnaire screening and allergen testing were defined as 1, and those whose responses were negative for both of these categories were defined as 0.

All analyses were performed with SPSS, version 23.0 (IBM-SPSS Statistics Inc). The statistical significance level was set at a 2-sided *P* value <.05.

## Results

A total of 17 349 children were included in this survey, and 16 936 parents or guardians (97.6%) completed the questionnaires. After the exclusion of 736 children because of twin pregnancies and 1055 owing to missing information on maternal weight, the valid sample of this study consisted of 15 145 mother-children pairs (eFigure 1 in the Supplement). Of these, 8877 children (58.6%) were screened for allergic diseases.

The mean (SD) age of the 15 145 children was 7.7 (2.33) years (range, 3-14 years) with 7911 boys (52.2%) and 7234 girls (47.8%). The prevalence rates of the allergies were 20.2% for asthma/wheezing, 28.8% for allergic rhinitis, 38.8% for eczema, and 11.9% for food/drug allergy. Allergic diseases were more likely to occur in children with older parents (2783 [32.4%] vs 1874 [31.0%]), higher family income (¥6402 [72.1%] vs ¥3786 [60.4%]), family history of allergic diseases (3241 [36.5%] vs 581 [9.3%]), and mothers who smoked during pregnancy (128 [1.4%] vs 130 [2.1%]) (Table 1).

**Table 1.** Parental Characteristics

Parental characteristic^a^	No. (%)			*P* value
	All	Allergic diseases		
		No	Yes	
Maternal age at delivery, y				
≤24	3020 (20.6)	1524 (25.2)	1496 (17.4)	<.001
25-29	6967 (47.6)	2645 (43.8)	4322 (50.2)	
≥30	4657 (31.8)	1874 (31.0)	2783 (32.4)	
Paternal age at delivery, y				
≤24	1762 (11.7)	930 (14.9)	832 (9.4)	<.001
25-34	11 023 (73.0)	4306 (68.9)	6717 (75.8)	
≥35	2320 (15.4)	1101 (16.2)	1309 (14.8)	
Family income (RMB), ¥^b^				
<6000	4957 (32.7)	2482 (39.6)	2475 (27.9)	<.001
≥6000	10 188 (67.3)	3786 (60.4)	6402 (72.1)	
Parity (No. of previous deliveries)				
<1	10 552 (69.7)	4365 (69.6)	6187 (69.7)	.94
≥2	4593 (30.3)	1903 (30.4)	2690 (30.3)	
Maternal smoking during pregnancy				
No	14 887 (98.3)	6138 (97.9)	8749 (98.6)	.003
Yes	258 (1.7)	130 (2.1)	128 (1.4)	
Maternal alcohol intake during pregnancy				
No	14 833 (97.9)	6133 (97.8)	8700 (98.0)	.50
Yes	312 (2.1)	135 (2.2)	177 (2.0)	
Paternal smoking during pregnancy				
No	9789 (64.6)	4099 (65.4)	5690 (64.1)	.10
Yes	5356 (35.4)	2169 (34.6)	3187 (35.9)	
Family history of allergic disease				
No	11 323 (74.8)	5687 (90.7)	5636 (63.5)	<.001
Yes	3822 (25.2)	581 (9.3)	3241 (36.5)	

^a^ Data missing for some variables.

^b^ RMB, currency in circulation in China; ¥1 is equivalent to $1.

Table 2 reports the differences of maternal prepregnancy BMI and GWG between groups with and without childhood allergies. Prepregnancy BMI did not differ significantly between the 2 groups, while significant differences were observed in GWG. For example, the rate of GWG greater than 25.0 kg was 4.7% for children without allergic disease and 6% for those with allergic diseases (*P* < .001). The results were similar when analyzed in the allergen test subgroup (eTable 1 in the Supplement).

**Table 2.** GWG and Prepregnancy BMI Characteristics of the Study Population

Characteristic	No. (%)			*P* value^a^
	All (n = 15 145)	Allergic diseases		
		No (n = 6268)	Yes (n = 8877)	
Prepregnancy BMI				
Underweight (<18.5)	3037 (20.1)	1250 (19.9)	1787 (20.1)	.59
Normal (18.5-22.9)	10 150 (67.0)	4180 (66.7)	5970 (67.3)	
Overweight (23.0-24.9)	1323 (8.7)	569 (9.1)	754 (8.5)	
Obese (≥25.0)	635 (4.2)	269 (4.3)	366 (4.1)	
GWG, kg				
<10.0	2651 (17.5)	1221 (19.5)	1430 (16.1)	<.001
10.0-15.0	7603 (50.2)	3204 (51.1)	4399 (49.6)	
15.0-25.0.	4064 (26.8)	1547 (24.7)	2517 (28.4)	
≥25.0	827 (5.5)	296 (4.7)	531 (6.0)	
GWG relative to IOM recommendation^b^				
Below	4932 (32.6)	2265 (36.1)	2667 (30.0)	<.001
Within	5410 (35.7)	2182 (34.8)	3228 (36.4)	
Mildly above	2778 (18.3)	1081 (17.2)	1697 (19.1)	
Extremely above	2025 (13.4)	740 (11.8)	1285 (14.5)	

Abbreviations: BMI, body mass index (calculated as weight in kilograms divided by height in meters squared); GWG, gestational weight gain; IOM, Institute of Medicine.

^a^ A χ^2^ test was performed on categorical variables to compare children with allergic disease with those without allergic disease.

^b^ Below IOM indicates below the range recommended by the IOM; within IOM, within the range recommended by the IOM; mildly above IOM, range from the IOM-recommended threshold to the median of the remaining values; and extremely above IOM, range from the median to the maximum.

The findings reported in Table 3 suggest that maternal prepregnancy BMI was not associated with any childhood allergies. With regard to absolute GWG, compared with GWG 10 to 15 kg, GWG less than 10 kg was associated with a decreased risk of overall allergies (adjusted PR, 0.93; 95% CI, 0.89-0.97; *P* < .001) and lower risks were shown for asthma/wheezing (10%), allergic rhinitis (9%), and eczema (11%). By contrast, GWG 15 to 25 kg was associated with an increased risk of childhood allergies (adjusted PR, 1.07; 95% CI, 1.04-1.10; *P* < .001). Moreover, GWG greater than or equal to 25 kg was associated with higher odds of childhood overall allergies (adjusted PR, 1.11; 95% CI, 1.05-1.17; *P* < .001) than for GWG 15 to 25 kg (adjusted PR, 1.07; 95% CI, 1.04-1.10; *P* < .001). In the analyses of the corresponding 4 individual diseases, increased risks were noted for GWG 15 to 25 kg for asthma/wheezing (13%), allergic rhinitis (13%), eczema (9%), and food/drug allergy (13%). In the GWG greater than 25 kg cohort, increased risks were also noted for asthma/wheezing (22%), allergic rhinitis (14%), eczema (15%), and food/drug allergy (23%).

**Table 3.** Associations of Maternal Prepregnancy BMI and GWG With the Risk of Childhood Allergic Diseases

Characteristic	95% CI												
	Allergic diseases		Asthma/wheezing			Allergic rhinitis			Eczema			Food/drug allergy	
	Crude	Adjusted^a^	Crude	Adjusted^a^	Crude		Adjusted^a^	Crude		Adjusted^a^	Crude		Adjusted^a^
**Prepregnancy BMI**^b^													
<18.5	1.00 (0.97-1.04)	1.00 (0.97-1.03)	0.95 (0.87-1.03)	0.95 (0.88-1.03)	1.01 (0.95-1.07)		1.01 (0.95-1.07)	1.03 (0.98-1.07)		1.03 (0.98-1.07)	1.09 (0.98-1.20)		1.09 (0.98-1.21)
*P* value	.98	.94	.19	.24	.77		.86	.28		.30	.10		.10
18.5-22.9	1 [Reference]	1 [Reference]	1 [Reference]	1 [Reference]	1 [Reference]		1 [Reference]	1 [Reference]		1 [Reference]	1 [Reference]		1 [Reference]
≥23.0	0.97 (0.93-1.01)	0.98 (0.94-1.02)	1.07 (0.98-1.18)	1.04 (0.96-1.13)	0.95 (0.88-1.02)		0.96 (0.89-1.03)	0.98 (0.92-1.04)		0.98 (0.92-1.04)	1.00 (0.88-1.13)		1.01 (0.89-1.14)
*P* value	.19	.36	.15	.36	.15		.26	.49		.42	.98		.89
*P* value for trend	.41	.66	.10	.10	.36		.48	.09		.35	.24		.25
**GWG, kg**^b^													
<10.0	0.93 (0.90-0.97)^c^	0.93 (0.89-0.97)^c^	0.91 (0.84-0.99)^c^	0.90 (0.82-0.98)^c^	0.92 (0.86-0.99)^c^		0.91 (0.85-0.98)^c^	0.89 (0.84-0.94)^c^		0.89 (0.84-0.94)^c^	0.95 (0.85-1.06)		0.93 (0.83-1.05)
*P* value	.001	<.001	.03	.01	.02		.01	<.001		<.001	.36		.25
10.0-15.0	1 [Reference]	1 [Reference]	1 [Reference]	1 [Reference]	1 [Reference]		1 [Reference]	1 [Reference]		1 [Reference]	1 [Reference]		1 [Reference]
15.0-25.0	1.07 (1.04-1.10)^c^	1.07 (1.04-1.10)^c^	1.14 (1.07-1.22)^c^	1.13 (1.06-1.21)^c^	1.13 (1.07-1.19)^c^		1.13 (1.07-1.19)^c^	1.09 (1.04-1.14)^c^		1.09 (1.04-1.13)^c^	1.14 (1.04-1.25)^c^		1.13 (1.02-1.24)^c^
*P* value	<.001	<.001	<.001	.02	<.001		<.001	<.001		<.001	.007		.02
≥25.0	1.11 (1.05-1.17)^c^	1.11 (1.05-1.17)^c^	1.22 (1.08-1.38)^c^	1.22 (1.08-1.38)^c^	1.14 (1.04-1.26)^c^		1.14 (1.03-1.26)^c^	1.15 (1.07-1.24)^c^		1.15 (1.07-1.24)^c^	1.23 (1.03-1.46)^c^		1.21 (1.01-1.44)^c^
*P* value	<.001	<.001	.001	.001	.007		.01	<.001		<.001	.02		.04
*P* value for trend	<.001	<.001	<.001	.001	<.001		<.001	<.001		<.001	.002		.004

Abbreviations: BMI, body mass index (calculated as weight in kilograms divided by height in meters squared); GWG, gestational weight gain; PR, prevalence ratio.

^a^ Adjusted for maternal age at delivery, paternal age at delivery, family income, parity, maternal smoking during pregnancy, maternal alcohol intake during pregnancy, paternal smoking during pregnancy, and family history of allergic disease.

^b^ Analyses were mutually adjusted for maternal prepregnancy BMI and gestational weight gain.

^c^ Statistically significant findings.

Table 4 reports the association between maternal GWG based on IOM guideline and childhood allergies. In mothers with GWG below the IOM guideline, the risk of overall childhood allergy was lower than the other GWG cohorts (adjusted PR, 0.91; 95% CI, 0.88-0.94; *P* < .001). For individual allergic diseases, reduced risks were noted for asthma/wheezing (13%), allergic rhinitis (11%), eczema (14%), and food/drug allergy (15%). An increased risk of overall childhood allergies was associated with maternal GWG above the IOM guideline (adjusted PR, 1.04; 95% CI, 1.01-1.07; *P* = .02). Furthermore, the GWG cohort extremely above the IOM guideline (adjusted PR, 1.07; 95% CI, 1.02-1.11; *P* = .002) was associated with higher risk compared with GWG mildly above the IOM guideline (adjusted PR, 1.02; 95% CI, 0.98-1.06; *P* = .28). Similar trends were observed in individual childhood allergic diseases: GWG extremely above the IOM guideline was associated with increased risks of asthma/wheezing (12%), allergic rhinitis (9%), and eczema (5%). A further increase in GWG (ie, extremely above the IOM guideline) was associated with increased risks for asthma/wheezing (19%), allergic rhinitis (11%), and eczema (10%). The analyses were evaluated in the allergen test subgroup and, when further stratified by children’s age group, the results were broadly consistent (eTable 2 and eTable 3 in the Supplement).

**Table 4.** Associations of IOM Recommendation–Based GWG With the Risk of Childhood Allergic Diseases

Characteristic	95% CI									
	Allergic diseases		Asthma/wheezing		Allergic rhinitis		Eczema		Food/drug allergy	
	Crude	Adjusted^a^	Crude	Adjusted^a^	Crude	Adjusted^a^	Crude	Adjusted^a^	Crude	Adjusted^a^
**GWG relative to IOM recommendation I**^**b**^										
Below IOM	0.91 (0.88-0.94)^**c**^	0.91 (0.88-0.94)^**c**^	0.87 (0.81-0.94)^**c**^	0.87 (0.80-0.93)^**c**^	0.89 (0.84-0.95)^**c**^	0.89 (0.84-0.94)^**c**^	0.86 (0.83-0.91)^**c**^	0.86 (0.82-0.91)^**c**^	0.85 (0.77-0.94)^**c**^	0.85 (0.77-0.94)^**c**^
*P* value	<.001	<.001	<.001	<.001	<.001	<.001	<.001	<.001	.002	.002
Within IOM	1 [Reference]	1 [Reference]	1 [Reference]	1 [Reference]	1 [Reference]	1 [Reference]	1 [Reference]	1 [Reference]	1 [Reference]	1 [Reference]
Above IOM	1.04 (1.01-1.07)^**c**^	1.04 (1.01-1.07)^**c**^	1.12 (1.04-1.19)^**c**^	1.12 (1.04-1.20)^**c**^	1.09 (1.03-1.15) ^**c**^	1.09 (1.03-1.15)^**c**^	1.05 (1.01-1.10)^**c**^	1.05 (1.01-1.10)^**c**^	1.09 (0.99-1.20)	1.08 (0.98-1.20)
*P* value	.01	.02	.002	.002	.002	.003	.03	.03	.09	.11
**GWG relative to IOM recommendation II**^**d**^										
Below IOM	0.91 (0.88-0.94)^**c**^	0.91 (0.88-0.94)^**c**^	0.87 (0.81-0.94)^**c**^	0.87 (0.80-0.93)^**c**^	0.89 (0.84-0.95)^**c**^	0.89 (0.84-0.94)^**c**^	0.86 (0.83-0.91)^**c**^	0.86 (0.82-0.91)^**c**^	0.85 (0.77-0.94)^**c**^	0.85 (0.77-0.94)^**c**^
*P* value	<.001	<.001	<.001	<.001	<.001	<.001	<.001	<.001	.002	.002
Within IOM	1 [Reference]	1 [Reference]	1 [Reference]	1 [Reference]	1 [Reference]	1 [Reference]	1 [Reference]	1 [Reference]	1 [Reference]	1 [Reference]
Mildly above IOM	1.02 (0.99-1.06)	1.02 (0.98-1.06)	1.07 (0.98-1.16)	1.06 (0.99-1.15)	1.08 (1.01-1.15)^**c**^	1.07 (1.01-1.14)^**c**^	1.02 (0.97-1.07)	1.01 (0.96-1.07)	1.07 (0.95-1.19)	1.06 (0.95-1.19)
*P* value	.21	.28	.12	.16	.02	.03	.50	.61	.28	.32
Extremely above IOM	1.06 (1.02-1.11)^**c**^	1.07 (1.02-1.11)^**c**^	1.18 (1.09-1.29)^**c**^	1.19 (1.10-1.30)^**c**^	1.10 (1.03-1.18)^**c**^	1.11 (1.03-1.19)^**c**^	1.09 (1.04-1.15)^**c**^	1.10 (1.04-1.16)^**c**^	1.18 (0.99-1.27)	1.12 (0.99-1.27)
*P* value	.002	.002	<.001	<.001	.006	.005	.001	.001	.08	.08

Abbreviations: GWG, gestational weight gain; IOM, Institute of Medicine; PR, prevalence ratio.

^a^ Adjusted for maternal age at delivery, paternal age at delivery, family income, parity, maternal smoking during pregnancy, maternal alcohol intake during pregnancy, maternal prepregnancy body mass index, paternal smoking during pregnancy, and family history of allergic disease.

^b^ Below IOM, below the range recommended by the IOM; Within IOM, within the range recommended by the IOM; Above IOM, above the range recommended by the IOM.

^c^ Statistically significant findings.

^d^ Below IOM indicates below the range recommended by the IOM; within IOM, within the range recommended by the IOM; mildly above IOM, range from the IOM-recommended threshold to the median of the remaining values; and extremely above IOM, range from the median to the maximum.

Table 5 presents the stratified analysis based on different maternal prepregnancy BMI levels. A GWG below the IOM guideline was associated with a lower risk of overall offspring allergies in prepregnancy underweight mothers (adjusted PR, 0.93; 95% CI, 0.87-0.98; *P* = .01), and the risk was further lowered in normal prepregnancy weight mothers (adjusted PR, 0.91; 95% CI, 0.88-0.95; *P* < .001). As for individual allergic diseases, GWG below the recommendation was associated with lowered risks for asthma/wheezing (13%), allergic rhinitis (12%), eczema (16%), and food/drug allergy (8%) when mothers were within the normal weight range before pregnancy. The lower risk was also observed in mothers in the prepregnancy underweight group for most cases, while the risk was not reduced among prepregnancy overweight/obese mothers. Extremely excessive GWG was associated with an elevated risk of childhood overall allergies in those whose mothers were overweight/obese before pregnancy (adjusted PR, 1.12; 95% CI, 1.03-1.22; *P* = .009). Gestational weight gain extremely above the IOM guideline in women who were overweight/obese before pregnancy was associated with the highest risk of childhood asthma/wheezing (adjusted PR, 1.42; 95% CI, 1.16-1.74; *P* = .001), allergic rhinitis (adjusted PR, 1.32; 95% CI, 1.12-1.56; *P* = .001), and eczema (adjusted PR, 1.24; 95% CI, 1.08-1.41; *P* = .002). Among 3 maternal prepregnancy BMI groups, GWG above the IOM-recommended threshold showed a higher prevalence of childhood allergic diseases, and the higher GWG corresponded with the higher prevalence of childhood allergies (eFigure 2 in the Supplement). However, the interaction between prepregnancy BMI and GWG on childhood allergies was nonsignificant (*P* = .52 for interaction) (eTable 4 in the Supplement). When stratified analyses were performed in preschool-aged and primary school–aged children, the results were generally consistent (eTable 5 in the Supplement).

**Table 5.** Associations of IOM Recommendation–Based GWG With Prevalence of Childhood Allergic Diseases Stratified by Maternal Prepregnancy BMI

Characteristic	95% CI									
	Allergic diseases		Asthma/wheezing		Allergic rhinitis		Eczema		Food/drug allergy	
	Crude	Adjusted^a^	Crude	Adjusted^a^	Crude	Adjusted^a^	Crude	Adjusted^a^	Crude	Adjusted^a^
**Maternal prepregnancy BMI <18.5**										
Below IOM^b^	0.91 (0.85-0.98)^c^	0.93 (0.87-0.98)^c^	0.81 (0.69-0.95)^c^	0.80 (0.68-0.94)^c^	0.89 (0.79-1.00)^c^	0.88 (0.78-1.00)^c^	0.90 (0.82-0.98)^c^	0.90 (0.82-0.99)^c^	0.90 (0.73-1.10)	0.89 (0.73-1.10)
*P* value	.01	.01	.01	.007	.05	.05	.02	.02	.30	.29
Within IOM^d^	1 [Reference]	1 [Reference]	1 [Reference]	1 [Reference]		1 [Reference]	1 [Reference]	1 [Reference]	1 [Reference]	1 [Reference]
Mildly above IOM^e^	1.05 (0.96-1.15)	1.03 (0.94-1.11)	1.16 (0.95-1.41)	1.14 (0.93-1.40)	1.17 (1.01-1.35)^c^	1.15 (0.99-1.34)	1.03 (0.91-1.17)	1.02 (0.90-1.16)	1.23 (0.95-1.61)	1.22 (0.94-1.60)
*P* value	.28	.63	.14	.20	.04	.06	.64	.72	.16	.14
Extremely above IOM^f^	1.07 (0.97-1.18)	1.07 (0.99-1.16)	1.07 (0.86-1.33)	1.08 (0.86-1.35)	1.14 (0.97-1.34)	1.12 (0.95-1.32)	1.11 (0.98-1.25)	1.09 (0.96-1.24)	1.30 (1.00-1.70)	1.26 (0.96-1.67)
*P* value	.16	.10	.57	.52	.11	.17	.10	.17	.05	.10
**Maternal prepregnancy BMI 18.5-22.9**										
Below IOM^b^	0.90 (0.86-0.93)^c^	0.91 (0.88-0.95)^c^	0.87 (0.80-0.95)^c^	0.87 (0.80-0.95)^c^	0.88 (0.83-0.95)^c^	0.88 (0.82-0.94)^c^	0.84 (0.80-0.89)^c^	0.84 (0.80-0.89)^c^	0.83 (0.73-0.94)^c^	0.92 (0.80-1.04)^c^
*P* value	<.001	<.001	.002	.02	<.001	<.001	<.001	<.001	.003	.003
Within IOM^d^	1 [Reference]	1 [Reference]	1 [Reference]	1 [Reference]	1 [Reference]	1 [Reference]	1 [Reference]	1 [Reference]	1 [Reference]	1 [Reference]
Mildly above IOM^e^	1.02 (0.98-1.07)	1.01 (0.97-1.05)	1.04 (0.94-1.14)	1.02 (0.92-1.13)	1.08 (1.00-1.17)^c^	1.08 (1.00-1.16)	1.02 (0.96-1.08)	1.01 (095-1.08)	1.10 (0.96-1.26)	122 (0.94-1.60)
*P* value	.36	.73	.50	.68	.04	.06	.58	.74	.17	.28
Extremely above IOM^f^	1.05 (1.00-1.11)	1.03 (0.99-1.08)^c^	1.17 (1.05-1.31)^c^	1.17 (1.04-1.31)^c^	1.06 (0.96-1.16)	1.06 (0.97-1.16)	1.08 (1.01-1.16)^c^	1.08 (1.01-1.16)^c^	1.05 (0.89-1.25)	1.26 (0.96-1.67)
*P* value	.06	.18	.005	.008	.24	.25	.03	.03	.88	.10
**Maternal prepregnancy BMI >23.0**										
Below IOM^b^	0.91 (0.78-1.06)	0.89 (0.78-1.02)	0.92 (0.68-1.24)	0.92 (0.67-1.25)	0.85 (0.66-1.11)	0.87 (0.67-1.14)	0.85 (0.68-1.05)	0.84 (0.68-1.05)	0.94 (0.64-1.37)	0.91 (0.62-1.35)
*P* value	.20	.10	.57	.59	.23	.31	.14	.13	.73	.65
Within IOM^d^	1 [Reference]	1 [Reference]	1 [Reference]	1 [Reference]	1 [Reference]	1 [Reference]	1 [Reference]	1 [Reference]	1 [Reference]	1 [Reference]
Mildly above IOM^e^	1.06 (0.96-1.18)	1.05 (0.96-1.14)	1.18 (0.96-1.46)	1.21 (0.97-1.50)	1.05 (0.87-1.25)	1.07 (0.89-1.28)	1.08 (0.94-1.25)	1.09 (0.95-1.26)	0.85 (0.62-1.16)	0.89 (0.65-1.23)
*P* value	.27	.34	.12	.10	.62	.51	.28	.23	.10	.48
Extremely above IOM^f^	1.16 (1.05-1.27)^c^	1.12 (1.03-1.22)^c^	1.37 (1.13-1.67)^c^	1.42 (1.16-1.74)^c^	1.28 (1.09-1.51)^c^	1.32 (1.12-1.56)^c^	1.22 (1.07-1.39)^c^	1.24 (1.08-1.41)^c^	1.16 (0.88-1.53)	1.22 (0.92-1.62)
*P* value	.003	.009	.002	.001	.003	.001	.004	.002	.93	.16

Abbreviations: BMI, body mass index; GWG, gestational weight gain; IOM, Institute of Medicine; PR, prevalence ratios.

^a^ Adjusted for maternal age at delivery, paternal age at delivery, family income, parity, maternal smoking during pregnancy, maternal alcohol intake during pregnancy, maternal prepregnancy body mass index, paternal smoking during pregnancy, family history of allergic disease.

^b^ Below the range recommended by the IOM.

^c^ Statistically significant findings.

^d^ Within the range recommended by the IOM.

^e^ The range is from the IOM-recommended threshold to the median of the remaining values.

^f^ The range is from the median to the maximum.

## Discussion

To our knowledge, this is the first study exploring the association of maternal prepregnancy BMI and GWG with childhood allergies in a low-income country. Common allergic diseases, including asthma/wheezing, allergic rhinitis, eczema, and food/drug allergy, were considered. An association between maternal prepregnancy BMI levels and all childhood allergic diseases was not established. Excessive GWG was associated with an increased risk of childhood asthma/wheezing, allergic rhinitis, and eczema. The risk appeared to be the highest among pregnant women who were overweight/obese before pregnancy. In mothers with low or normal prepregnancy weight, low GWG was significantly associated with a decreased risk of the 4 childhood allergies evaluated in most cases, but the significance disappeared in mothers who were overweight/obese before pregnancy. A similar magnitude of association was noted in analysis of the allergen test subgroup.

Many studies have focused on the association between maternal BMI around pregnancy and childhood allergies, while few have examined GWG as a potential risk. In 2013, 3 birth cohort studies initially suggested that excessive GWG was associated with an increased risk of asthma and wheezing in children.^10,21,26^ Since then, the role of maternal weight gain in childhood allergies became an increasing concern. A national study in the US published in 2017 similarly reported that extremely high GWG (>25 kg) was associated with higher odds of childhood asthma.^17^ Another study in the US further noted an association between excessive GWG (≥20 kg) and the risk of childhood atopic dermatitis.^24^ The consistent results were also observed in our study that excessive GWG was associated with asthma/wheezing, allergic rhinitis, and eczema in children, and the association demonstrated a corresponding increase between the GWG and risk of childhood allergies.

Excess GWG combined with prepregnancy overweight/obesity appeared to have the most significant association, in most categories of the primary school–aged children. Similarly, the latest US cohort study reported that higher GWG combined with prepregnancy BMI greater than 25 was associated with a higher risk for atopic dermatitis.^24^ We did not find a significant interaction between GWG and maternal prepregnancy BMI, which was also reported in previous studies.^24,25^ In contrast to excessive GWG, we found that offspring of mothers with low GWG had a lower risk of childhood allergies in most cases, especially in primary school–aged children. However, the significance disappeared when mothers were categorized as overweight/obese before pregnancy. Previous studies focused less on the low GWG, and their results were divergent. In general, 2 studies found that low GWG was associated with an increased risk of childhood asthma,^17,35^ while 4 studies did not support a significant association between low GWG and childhood asthma,^25^ wheezing,^20,36^ and atopic dermatitis.^24^ The association is not exclusively explained; since different covariables and population ages could all contribute to divergences, the conflicting results warrant further study.

Previous studies reported that maternal prepregnancy weight status seemed to be associated with childhood allergies, mainly for asthma and wheezing; however, there are some discrepancies.^8,9,10,16,17,18,19,20,22,37^ A Dutch birth cohort based on children aged 1 to 4 years suggested that maternal prepregnancy obesity was associated with an increased risk of wheezing, mainly in mothers with a history of atopy.^10^ However, a Northern Finland birth cohort demonstrated that an increased risk of childhood wheezing and asthma was observed only in children without a parental history of atopy.^22^ In contrast with those 2 studies, a cohort study in the US with participants aged 16 years or younger showed no association between maternal prepregnancy BMI and childhood atopic dermatitis.^24^ Similar to our results, no apparent association was observed between maternal prepregnancy weight status and any of the childhood allergies examined. There is a paucity of data examining other allergic symptoms. Different ethnic groups contributing to different body fat distribution, Eastern and Western living conditions, and lower obesity rates in low-income countries may lead to discrepancies.^38^ Since this study is, to our knowledge, the first to be carried out in a Chinese population, it provides useful information for further investigation of prepregnancy BMI and GWG and childhood allergic diseases in diverse regions.

The mechanism by which maternal body weight management is associated with childhood allergic diseases has not been elucidated, but may be a factor in specific inflammatory states. An abnormal rate of GWG contributed to the metabolic and hormonal environment of the developing fetus has been reported to alter with elevated concentrations of inflammatory markers, for instance, C-reactive protein.^39^ Regardless of maternal baseline BMI, excessive GWG is associated with increased inflammation,^39^ which may explain our different observations of prepregnancy BMI and GWG in different childhood allergic diseases. Moreover, the pathogenesis of allergic diseases might be associated with excessive GWG and prepregnancy overweight/obesity through exposure of the fetus to suboptimal dietary patterns.^40^ High-fat diets that lead to excess GWG were more likely to lower serum vitamin D levels.^41^ Vitamin D deficiency during pregnancy has proved to be associated with an increased risk of childhood allergies.^42^ In addition, dietary choices during pregnancy have been linked to allergic diseases in children.^43,44^ Furthermore, GWG leads to constant changes in the mother's intestinal microorganisms, and the relationship between atopic diseases and microbial community is affected by the underlying bidirectional mechanism.^45^

### Limitations

This study has several limitations. First, recall bias on maternal weight before and during pregnancy is inevitable, and correspondingly, misclassification could not be avoided. However, as the 1-child policy was once widely enforced in China, mothers were carefully aware of their condition during this once-in-a-lifetime opportunity. In addition, the stratified analyses in preschool and primary school children showed broadly consistent changes, which to some extent revealed that misclassification caused by recall bias could be limited and nondifferential. Second, allergic diseases were not physician diagnosed but were based on parent-reported data, which may contribute to the misclassification of allergic diseases. Nevertheless, the ISAAC questionnaire was adopted, which is internationally recognized as having good reliability and validity. Meanwhile, we checked the validity of parent-reported allergy through the subgroup of children who ever had allergen testing, with the findings showing that children without any allergic disease screened by the ISAAC questionnaire were also reported having negative results on allergen testing. In addition, causality cannot be determined in cross-sectional studies. While information about mothers before and after childbirth was chronologically linked to their children's health status, an association, but not cause, could be noted.

## Conclusions

In this relatively large epidemiologic study, excessive GWG appeared to be associated with an increased risk of childhood allergies. The more gestational weight a mother gained, the higher the risk for childhood allergies appeared to be. In particular, prepregnancy overweight/obesity status may be a modifying factor in excessive weight gain, although there appears to be no interaction effect on childhood allergies. Gestational weight gain below the IOM guideline appeared to be associated with a decreased risk of childhood allergies in women with normal prepregnancy weight and women who were underweight. In view of the high prevalence of childhood allergic diseases and their effect on the health of children, it is recommended that women maintain appropriate weight before pregnancy and prevent excessive GWG as potential prevention measures for allergic diseases in their children.

## Notes

Supplement. **eTable 1.** GWG and Pre-Pregnancy BMI Characteristics of the Allergen Test Subgroup **eTable 2.** Associations of IOM Recommendation-Based GWG With the Risk of Childhood Allergic Diseases in the Allergen Test Subgroup **eTable 3.** Associations of IOM Recommendation-Based GWG With the Risk of Four Single Allergic Diseases Stratified by Children’s Age Group **eTable 4.** Associations of Maternal GWG With Childhood Allergies, According to Maternal Pre-Pregnancy BMI **eTable 5.** Associations of IOM Recommendation-Based GWG With the Risk of Four Single Allergic Diseases, Stratified by Children’s Age Group and Maternal Pre-Pregnancy BMI **eFigure 1.** Flow Chart of Participants Included for Analysis **eFigure 2.** Maternal GWG and the Prevalence of Childhood Allergic Diseases According to Pre-Pregnancy BMI **eAppendix.** Childhood Allergy Questionnaire
