# Supplementary material for: Association of Maternal Prepregnancy Weight and Gestational Weight Gain With Children’s Allergic Diseases
Source: JAMA Netw Open. 2020 Sep 2;3(9):e2015643. doi: 10.1001/jamanetworkopen.2020.15643 (PMC7489810; doi:10.1001/jamanetworkopen.2020.15643)
Supplement: Supplement. — eTable 1. GWG and Pre-Pregnancy BMI Characteristics of the Allergen Test Subgroup eTable 2. Associations of IOM Recommendation-Based GWG With the Risk of Childhood Allergic Diseases in the Allergen Test Subgroup eTable 3. Associations of IOM Recommendation-Based GWG With the Risk of Four Single Allergic Diseases Stratified by Children’s Age Group eTable 4. Associations of Maternal GWG With Childhood Allergies, According to Maternal Pre-Pregnancy BMI eTable 5. Associations of IOM Recommendation-Based GWG With the Risk of Four Single Allergic Diseases, Stratified by Children’s Age Group and Maternal Pre-Pregnancy BMI eFigure 1. Flow Chart of Participants Included for Analysis eFigure 2. Maternal GWG and the Prevalence of Childhood Allergic Diseases According to Pre-Pregnancy BMI eAppendix. Childhood Allergy Questionnaire [file jamanetwopen-e2015643-s001.pdf]

Supplementary Online Content

Chen Y, Zhu J, Lyu J, et al. Association of maternal prepregnancy weight and gestational weight gain with children's allergic diseases. *JAMA Netw Open*. 2020;3(9):e2015643. doi:10.1001/jamanetworkopen.2020.15643

eTable 1. GWG and Pre-Pregnancy BMI Characteristics of the Allergen Test Subgroup

eTable 2. Associations of IOM Recommendation-Based GWG With the Risk of Childhood Allergic Diseases in the Allergen Test Subgroup

eTable 3. Associations of IOM Recommendation-Based GWG With the Risk of Four Single Allergic Diseases Stratified by Children's Age Group

eTable 4. Associations of Maternal GWG With Childhood Allergies, According to Maternal Pre-Pregnancy BMI

eTable 5. Associations of IOM Recommendation-Based GWG With the Risk of Four Single Allergic Diseases, Stratified by Children's Age Group and Maternal Pre-Pregnancy BMI

eFigure 1. Flow Chart of Participants Included for Analysis

eFigure 2. Maternal GWG and the Prevalence of Childhood Allergic Diseases According to Pre-Pregnancy BMI

eAppendix. Childhood Allergy Questionnaire

This supplementary material has been provided by the authors to give readers additional information about their work.

© 2020 Chen Y et al. *JAMA Network Open.*

**eTable 1 GWG and pre-pregnancy BMI characteristics of the allergen test subgroup**				
	All (n = 8,235)	Allergen Test Subgroup		*p*-value
		No (n =5,637)	Yes (n =2,598)	
**Pre-pregnancy BMI, kg/m^2, no (%)**				.59
Underweight (< 18.5)	1677 (20.4%)	1132 (20.1%)	545 (21.0%)	
Normal (18.5-22.9)	5467 (66.4%)	3761 (66.7%)	1706 (65.7%)	
Overweight (23.0-24.9)	729 (8.9%)	504 (8.9%)	225 (8.7%)	
Obese (≥ 25.0)	362 (4.4%)	240 (4.3%)	122 (4.7%)	
**GWG, kg, no (%)**				**<.001**
< 10.0	1494 (18.1%)	1093 (19.4%)	401 (15.4%)	
10.0-15.0	4167 (50.6%)	2880 (51.1%)	1287 (49.5%)	
15.0-25.0.	2148 (26.1%)	1394 (24.7%)	754 (29.0%)	
≥ 25.0	426 (5.2%)	270 (4.8%)	156 (6.0%)	
**GWG relative to IOM recommendation, no (%)**				**<.001**
Below IOM	2786 (33.8%)	2013 (35.7%)	773 (29.8%)	
Within IOM	2904 (35.3%)	1984 (35.2%)	920 (35.4%)	
Mild above IOM	1498 (18.2%)	981 (17.4%)	517 (19.9%)	
Extremely above IOM	1047 (12.7%)	659 (11.7%)	388 (14.9%)	

Abbreviations: BMI, body mass index; GWG, gestational weight gain; IOM, Institute of Medicine.

$\chi2$ test performed on categorical variables to compared offspring with allergic disease to offspring without allergic disease.

Below IOM, below the range recommended by the IOM; Within IOM, within the range recommended by the IOM; Mild above IOM, the range is from the IOM recommended threshold to the median of the remaining values; Extremely above IOM, the range is from the median to the maximum.

© 2020 Chen Y et al. *JAMA Network Open.*

eTable 2 Associations of IOM recommendation-based GWG with the risk of childhood allergic diseases in the Allergen Test Subgroup

	Allergen Test Subgroup			
	Crude PR (95% CI)	*P*-value	Adjusted PR (95% CI)	*P*-value
**GWG relative to IOM recommendation I [a]**				
Below IOM	**0.88 (0.81-0.95)**	**.001**	**0.86 (0.80-0.94)**	**<.001**
Within IOM	ref		ref	
Above IOM	**1.12 (1.04-1.21)**	**.002**	**1.10 (1.02-1.19)**	**.01**
**GWG relative to IOM recommendation II [b]**				
Below IOM	**0.88 (0.81-0.95)**	**.001**	**0.86 (0.80-0.94)**	**<.001**
Within IOM	ref		ref	
Mild above IOM	1.09 (1.00-1.19)	.06	1.07 (0.98-1.17)	.13
Extremely above IOM	**1.17 (1.06-1.29)**	**.001**	**1.15 (1.05-1.27)**	**.004**

Abbreviations: GWG, gestational weight gain; IOM, Institute of Medicine; CI, confidence interval; PR, prevalence ratios.

Adjusted for maternal age at delivery, paternal age at delivery, family income, parity, maternal smoking during pregnancy, maternal alcohol intake during pregnancy, maternal pre-pregnancy body mass index, paternal smoking during pregnancy, family history of allergic disease.

[a] Below IOM, below the range recommended by the IOM; Within IOM, within the range recommended by the IOM; Above IOM, above the range recommended by the IOM.

[b] Below IOM, below the range recommended by the IOM; Within IOM, within the range recommended by the IOM; Mild above IOM, the range is from the IOM recommended threshold to the median of the remaining values; Extremely above IOM, the range is from the median to the maximum.

Significant results are shown in bold font.

© 2020 Chen Y et al. *JAMA Network Open.*

eTable 3 Associations of IOM recommendation-based GWG with the risk of four single allergic diseases stratified by children's age group

	**Asthma/Wheeze**		**Allergic rhinitis**		**Eczema**		**Food/ drug allergy**	
	Crude PR (95% CI)	Adjusted PR (95% CI)	Crude PR (95% CI)	Adjusted PR (95% CI)	Crude PR (95% CI)	Adjusted PR (95% CI)	Crude PR (95% CI)	Adjusted PR (95% CI)
**Preschool Children**								
**GWG I [a]**								
Below IOM	0.99 (0.89-1.11)	0.99 (0.89-1.12)	1.00 (0.91-1.10)	1.00 (0.90-1.10)	**0.92 (0.86-0.99)**	**0.92 (0.86-0.99)**	0.96 (0.82-1.13)	0.96 (0.82-1.13)
p-value	.91	.92	.96	.95	**.02**	**.02**	.65	.63
Within IOM	ref	ref	ref	ref	ref	ref	ref	ref
Above IOM	**1.19 (1.07-1.32)**	**1.20 (1.08-1.34)**	**1.12 (1.02-1.23)**	**1.13 (1.03-1.24)**	1.02 (0.96-1.09)	1.02 (0.96-1.09)	1.05 (0.89-1.22)	1.07 (0.91-1.25)
p-value	**.001**	**.001**	**.02**	**.01**	.56	.47	.58	.43
**GWG II [b]**								
Below IOM	0.99 (0.89-1.11)	0.99 (0.89-1.12)	1.00 (0.91-1.10)	1.00 (0.90-1.10)	**0.92 (0.86-0.99)**	**0.92 (0.86-0.99)**	0.96 (0.82-1.13)	0.96 (0.82-1.13)
p-value	.91	.92	.96	.95	**.02**	**.02**	.65	.63
Within IOM	ref	ref	ref	ref	ref	ref	ref	ref
Mild above IOM	**1.16 (1.03-1.31)**	**1.16 (1.03-1.32)**	1.08 (0.97-1.21)	1.09 (0.97-1.22)	1.00 (0.93-1.08)	1.00 (0.93-1.08)	0.97 (0.80-1.17)	0.98 (0.81-1.19)
p-value	**.02**	**.02**	.16	.15	.99	.47	.72	.82
Extremely above IOM	**1.22 (1.07-1.39)**	**1.25 (1.10-1.42)**	**1.17 (1.04-1.31)**	**1.19 (1.06-1.34)**	1.04 (0.96-1.13)	1.05 (0.97-1.14)	1.15 (0.95-1.40)	1.18 (0.97-1.43)
p-value	**.002**	**.001**	**.008**	**.003**	.29	.20	.16	.10
**Primary School Children**								
**GWG I [a]**								

© 2020 Chen, Y, et al. *JAMA Network Open.*

Below IOM	**0.80 (0.73-0.88)**	**0.79 (0.72-0.87)**	**0.84 (0.79-0.91)**	**0.84 (0.78-0.90)**	**0.83 (0.78-0.89)**	**0.83 (0.78-0.89)**	**0.79 (0.70-0.90)**	**0.79 (0.69-0.90)**
p-value	**<.001**	**<.000**	**<.001**	**<.001**	**<.001**	**<.001**	**<.001**	**.001**
Within IOM	ref	ref	ref	ref	ref	ref	ref	ref
Above IOM	1.06 (0.97-1.16)	1.06 (0.96-1.16)	**1.07 (1.00-1.14)**	1.07 (1.00-1.14)	**1.07 (1.01-1.14)**	**1.07 (1.01-1.13)**	1.11 (0.98-1.26)	1.10 (0.97-1.24)
p-value	.20	.26	**.04**	.06	**.02**	**.03**	.09	.15
**GWG II [b]**								
Below IOM	**0.80 (0.73-0.88)**	**0.79 (0.72-0.87)**	**0.84 (0.79-0.91)**	**0.84 (0.78-0.90)**	**0.83 (0.78-0.89)**	**0.83 (0.78-0.89)**	**0.79 (0.70-0.90)**	**0.79 (0.69-0.90)**
p-value	**<.001**	**<.001**	**<.001**	**<.001**	**<.001**	**<.001**	**<.001**	**.001**
Within IOM	ref	ref	ref	ref	ref	ref	ref	ref
Mild above IOM	1.00 (0.90-1.12)	0.99 (0.89-1.11)	1.07 (1.00-1.16)	1.07 (0.99-1.15)	1.03 (0.96-1.11)	1.03 (0.96-1.10)	1.12 (0.98-1.29)	1.11 (0.96-1.28)
p-value	.99	.86	.07	.10	.74	.49	.11	.16
Extremely above IOM	**1.15 (1.02-1.28)**	**1.15 (1.02-1.29)**	1.07 (0.98-1.17)	1.06 (0.97-1.16)	**1.13 (1.05-1.21)**	**1.13 (1.05-1.21)**	1.09 (0.93-1.29)	1.07 (0.91-1.27)
p-value	**.02**	**.02**	.13	.17	**.001**	**.001**	.30	.41

Abbreviations: GWG, gestational weight gain; IOM, Institute of Medicine; PR, prevalence ratios; CI, confidence interval.

Adjusted for maternal age at delivery, paternal age at delivery, family income, parity, maternal smoking during pregnancy, maternal alcohol intake during pregnancy, paternal smoking during pregnancy, maternal pre-pregnancy body mass index, family history of allergic disease.

[a] Within IOM, within the range recommended by the IOM; Above IOM, above the range recommended by the IOM.

[b] Within IOM, within the range recommended by the IOM; Mild above IOM, the range is from the IOM recommended threshold to the median of the remaining values; Extremely above IOM, the range is from the median to the maximum.

Significant results are shown in bold font.

© 2020 Chen, Y, et al. *JAMA Network Open.*

**eTable 4 Associations of maternal GWG with childhood allergies, according to maternal pre-pregnancy BMI**							
	Maternal pre-pregnancy BMI, kg/m^2						
		< 18.5		18.5-22.9		>23	
	No of cases	PR (95% CI)	No of cases	PR (95% CI)	No of cases	PR (95% CI)	*p*-interaction
Maternal GWG							
<10	429	**0.93 (0.88-0,98)**	1696	0.94 (0.85-1.04)	531	**1.25 (1.08-1.46)**	
10-15	1494	ref	5124	ref	985	ref	.52
16-24	924	**1.05 (1.01-1.10)**	2776	**1.09 (1.02-1.16)**	362	**1.11 (1.01-1.23)**	
⩾25	195	**1.11 (1.04-1.19)**	552	1.03 (0.91-1.17)	80	**1.25 (1.08-1.46)**	

Abbreviations: BMI, body mass index; GWG, gestational weight gain; CI, confidence interval; PR, prevalence ratios.

Adjusted for maternal age at delivery, paternal age at delivery, family income, parity, maternal smoking during pregnancy, maternal alcohol intake during pregnancy, paternal smoking during pregnancy, family history of allergic disease.

Significant results are shown in bold font.

© 2020 Chen, Y, et al. *JAMA Network Open.*

eTable 5 Associations of IOM recommendation-based GWG with the risk of four single allergic diseases, stratified by children's age group and maternal pre-pregnancy BMI

	Asthma/Wheeze		Allergic rhinitis		Eczema		Food/ drug allergy	
	Crude PR (95% CI)	Adjusted PR (95% CI)	Crude PR (95% CI)	Adjusted PR (95% CI)	Crude PR (95% CI)	Adjusted PR (95% CI)	Crude PR (95% CI)	Adjusted PR (95% CI)
**Preschool Children**								
**Maternal pre-pregnancy BMI < 18.5**								
Below IOM	1.12 (0.87-1.44)	1.13 (0.86-1.43)	1.11 (0.90-1.38)	1.09 (0.88-1.35)	1.02 (0.89-1.18)	1.02 (0.89-1.18)	1.28 (0.91-1.79)	1.28 (0.91-1.80)
p-value	.37	.41	.32	.43	.74	.76	.16	.16
Within IOM	ref	ref	ref	ref	ref	ref	ref	ref
Mild above IOM	**1.48 (1.09-2.02)**	**1.42 (1.02-2.05)**	**1.33 (1.01-1.77)**	1.29 (0.96-1.72)	1.17 (0.96-1.41)	1.14 (0.94-1.39)	1.49 (0.93-2.38)	1.44 (0.88-2.34)
p-value	**.01**	**.04**	**.05**	.09	.12	.18	.10	.15
Extremely above IOM	1.13 (0.76-1.67)	1.12 (0.73-1.97)	1.25 (0.93-1.69)	1.30 (0.96-1.75)	1.10 (0.90-1.36)	1.14 (0.93-1.39)	1.42 (0.87-2.31)	1.52 (0.93-2.46)
p-value	.54	.60	.15	.09	.34	.22	.16	.09
**Maternal pre-pregnancy BMI 18.5- 22.9**								
Below IOM	0.97 (0.85-1.11)	0.97 (0.85-1.11)	0.97 (0.86-1.09)	0.97 (0.86-1.09)	**0.90 (0.83-0.98)**	**0.90 (0.83-0.98)**	0.92 (0.76-1.12)	0.92 (0.76-1.12)
p-value	.69	.68	.59	.59	**.01**	**<.001**	.41	0.42
Within IOM	ref	ref	ref	ref	ref	ref	ref	ref
Mild above IOM	1.08 (0.92-1.25)	1.07 (0.92-1.25)	1.05 (0.92-1.20)	1.05 (0.92-1.20)	0.96 (0.87-1.05)	0.96 (0.87-1.05)	0.87 (0.69-1.11)	0.89 (0.70-1.13)
p-value	.35	.38	.48	.47	.36	.35	.27	.33
Extremely above IOM	**1.27 (1.08-1.49)**	**1.28 (1.09-1.51)**	**1.19 (1.02-1.37)**	**1.20 (1.04-1.39)**	1.06 (0.96-1.17)	1.07 (0.96-1.18)	1.18 (0.86-1.45)	1.14 (0.88-1.48)

© 2020 Chen, Y, et al. *JAMA Network Open.*

eTable 5, cont								
p-value	**.004**	**.003**	**.02**	**.01**	.27	.22	.75	.31
**Maternal pre-pregnancy BMI > 23**								
Below IOM	0.76 (0.46-1.24)	0.79 (0.48-1.29)	0.88 (0.58-1.31)	0.91 (0.61-1.37)	0.72 (0.51-1.00)	0.73 (0.52-1.02)	0.44 (0.18-1.05)	**0.36 (0.14-0.97)**
p-value	.27	.34	.52	.66	.51	.07	.06	**.04**
Within IOM	ref	ref	ref	ref	ref	ref	ref	ref
Mild above IOM	1.20 (0.89-1.61)	1.25 (0.93-1.69)	0.99 (0.74-1.33)	1.04 (0.77-1.40)	1.02 (0.85-1.23)	1.04 (0.86-1.25)	0.91 (0.58-1.43)	0.94 (0.60-1.47)
p-value	.23	.10	.71	.79	.86	.68	.69	.77
Extremely above IOM	1.13 (0.85-1.51)	1.17 (0.87-1.56)	1.10 (0.84-1.42)	1.13 (0.86-1.47)	0.99 (0.83-1.19)	1.00 (0.84-1.20)	1.01 (0.68-1.52)	1.02 (0.68-1.53)
	.41	.30	50	.38	.92	1.00	.95	.92

© 2020 Chen, Y, et al. *JAMA Network Open.*

eTable 5 Associations of IOM recommendation-based GWG with the risk of four single allergic diseases, stratified by children's age group and maternal pre-pregnancy BMI

**Primary School Children**								
**Maternal pre-pregnancy BMI < 18.5**								
Below IOM	**0.65 (0.53-0.81)**	**0.63 (0.51-0.79)**	**0.80 (0.69-0.92)**	**0.79 (0.68-0.92)**	**0.82 (0.72-0.92)**	**0.82 (0.72-0.93)**	0.73 (0.56-0.95)	0.72 (0.56-0.94)
p-value	**<.001**	**<.001**	**.002**	**.003**	**.001**	**.002**	.02	.02
Within IOM	ref	ref	ref	ref	ref	ref	ref	ref
Mild above IOM	1.01 (0.79-1.30)	1.01 (0.78-1.31)	1.09 (0.92-1.30)	1.09 (0.92-1.30)	0.96 (0.81-1.13)	0.96 (0.81-1.14)	1.13 (0.82-1.54)	1.13 (0.82-1.54)
p-value	.92	.99	.30	.33	.62	.64	.46	.46
Extremely above IOM	1.03 (0.79-1.35)	1.02 (0.77-1.34)	1.09 (0.91-1.31)	1.05 (0.86-1.28)	1.11 (0.95-1.29)	1.07 (0.91-1.26)	1.24 (0.90-1.71)	1.15 (0.83-1.61)
p-value	.81	.91	.36	.64	.18	.40	.18	.41
**Maternal pre-pregnancy BMI 18.5- 22.9**								
Below IOM	**0.82 (0.73-0.92)**	**0.81 (0.72-0.91)**	**0.84 (0.78-0.92)**	**0.84 (0.77-0.91)**	**0.81 (0.75-0.88)**	**0.81 (0.75-0.88)**	**0.78 (0.66-0.91)**	**0.78 (0.66-0.91)**
p-value	**<.001**	**<.001**	**<.001**	**<.001**	**<.001**	**<.001**	**.002**	**.002**
Within IOM	ref	ref	ref	ref	ref	ref	ref	ref
Mild above IOM	1.00 (0.87-1.14)	0.98 (0.85-1.12)	**1.10 (1.00-1.20)**	1.09 (0.99-1.20)	1.06 (0.98-1.15)	1.05 (0.97-1.14)	**1.25 (1.05-1.48)**	**1.21 (1.02-1.44)**
p-value	.97	.75	**.04**	.07	.16	.25	**.01**	**.03**
Extremely above IOM	1.09 (0.94-1.27)	1.07 (0.92-1.25)	0.98 (0.87-1.11)	0.97 (0.86-1.10)	1.09 (0.99-1.20)	1.09 (0.99-1.21)	1.00 (0.80-1.26)	0.97 (0.76-1.23)
p-value	.26	.39	.80	.66	.05	.07	.98	.78
**Maternal pre-pregnancy BMI > 23**								
Below IOM	1.05 (0.71-1.55)	1.04 (0.70-1.55)	0.84 (0.59-1.18)	0.85 (0.60-1.20)	1.00 (0.75-1.34)	1.00 (0.74-1.34)	1.26 (0.82-1.96)	1.30 (0.82-2.05)
p-value	.80	.86	.31	.35	.98	.97	.20	.15

© 2020 Chen, Y, et al. *JAMA Network Open.*

Within IOM	ref	ref	ref	ref	ref	ref	ref	ref
Mild above IOM	1.17 (0.87-1.57)	1.18 (0.87-1.60)	1.09 (0.86-1.37)	1.08 (0.86-1.37)	1.18 (0.96-1.45)	1.19 (0.96-1.47)	0.81 (0.53-1.24)	0.88 (0.57-1.37)
p-value	.47	.28	.49	.50	.13	.12	.33	.57
Extremely above IOM	**1.58 (1.20-2.07)**	**1.65 (1.25-2.17)**	**1.42 (1.15-1.74)**	**1.46 (1.18-1.80)**	**1.46 (1.21-1.77)**	**1.52 (1.25-1.85)**	1.26 (0.86-1.84)	1.39 (0.94-2.06)
p-value	**<.001**	**<.001**	**.001**	**<.001**	**<.001**	**<.001**	.23	.10

Abbreviations: BMI, body mass index; IOM, Institute of Medicine; PR, prevalence ratios; CI, confidence interval.

[a] Adjusted for maternal age at delivery, paternal age at delivery, family income, parity, maternal smoking during pregnancy, maternal alcohol intake during pregnancy, maternal pre-pregnancy body mass index, paternal smoking during pregnancy, family history of allergic disease .

[b] Within IOM, within the range recommended by the IOM; Mild above IOM, the range is from the IOM recommended threshold to the median of the remaining values; Extremely above IOM, the range is from the median to the maximum. Significant results are shown in bold font.

© 2020 Chen, Y, et al. *JAMA Network Open.*

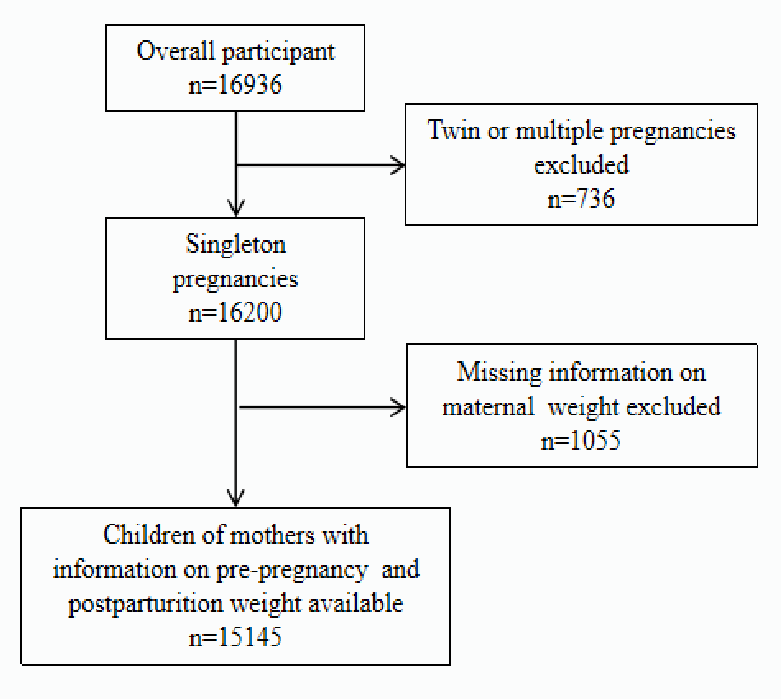

eFigure 1 Flow chart of participants included for analysis

© 2020 Chen, Y, et al. *JAMA Network Open.*

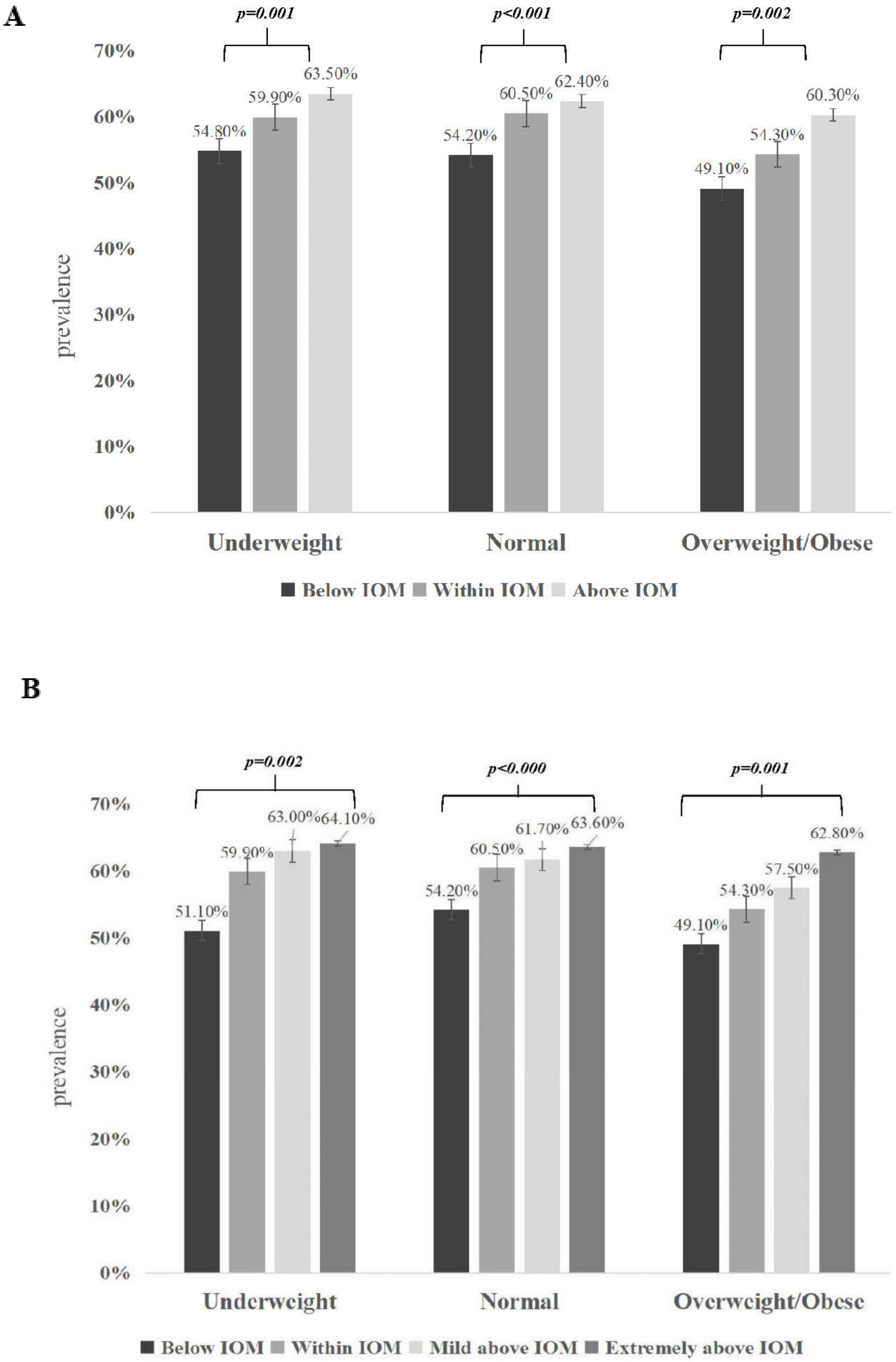

eFigure 2 Maternal GWG and the prevalence of childhood allergic diseases according to pre-pregnancy BMI

Abbreviation: BMI, body mass index; GWG, gestational weight gain; IOM, Institute of Medicine.

© 2020 Chen, Y, et al. *JAMA Network Open.*

[a] Underweight, pre-pregnancy body mass index below 18.5; Normal, pre-pregnancy body mass index between 18.5 and 23; Overweight/obese, pre-pregnancy body mass index above 23.

[b] Below IOM, below the range recommended by the IOM; Within IOM, within the range recommended by the IOM; Mild above IOM, the range is from the IOM recommended threshold to the median of the remaining values; Extremely above IOM, the range is from the median to the maximum.

© 2020 Chen, Y, et al. *JAMA Network Open.*

eAppendix. CHILDHOOD ALLERGY QUESTIONNAIRE

1.Has your child ever been diagnosed with asthma?

□ yes □ no

2.Has your child experienced any wheezing, breathing difficulties, or whistling in the chest in the last 12 months?

□ yes □ no

3.Has your child ever been diagnosed with allergic rhinitis?

□ yes □ no

4.Has your child ever sneezing, runny nose, stuffy, or itchy in the last 12 months not due to having a cold or flu?

□ yes □ no

4a. If YES, has this nose problem been accompanied by itchy–watery eyes?

□ yes □ no

5. In the past 12 months, has your child had “hay fever”?

□ yes □ no

6.Has your child had repeated skin itching or a rash on the bent side of the elbow or knee joint in the last 12 months?

□ yes □ no

7.Has your child ever been diagnosed with eczema?

□ yes □ no

8.Has your child had a history of food allergies (redness, swelling, rash, abdominal pain, etc. within 2 hours after eating)?

□ yes □ no

9.Has your child ever been diagnosed by a doctor with a drug allergy?

□ yes □ no

© 2020 Chen, Y, et al. *JAMA Network Open.*

[*] All questions were translated into Chinese when used.

[#] To identify ever asthma, the question was asked: “Has your child ever been diagnosed with asthma?”, and ever wheezing was defined by question: “Has your child experienced any wheezing, breathing difficulties or whistling in the chest in the last 12 months?”. Question utilized to identify ever allergic rhinitis was: “Has your child ever been diagnosed with allergic rhinitis?” and three questions was combined to determine current allergic rhinitis: “Has your child ever sneezing, runny nose, stuffy, itchy in the last 12 months not due to having a cold or flu?”, “If YES, has this nose problem been accompanied by itchy–watery eyes?”, “In the past 12 months, has your child had “hay fever”?”. Ever eczema was confirmed by question: “Has your child ever been diagnosed with eczema?” and current eczema was determined by “Has your child had repeated itching or rash on the bent side of the elbow or knee joint in the last 12 months?”. Food allergy was ascertained by the positive answer to question: “Does your child have a history of food allergies (redness, swelling, rash, abdominal pain, etc. within 2 hours after eating)?”. Drug allergy was determined by question: “Has your child ever been diagnosed by a doctor with a drug allergy?” The screened asthma/wheezing, allergic rhinitis and eczema were determined by ever diagnosis plus current symptoms. Children who were screened out one of above five types of allergies were considered to be screened positive for allergic diseases.

© 2020 Chen, Y, et al. *JAMA Network Open.*
